# Calcium oxalate crystal-induced secretome derived from proximal tubular cells, not that from distal tubular cells, induces renal fibroblast activation

**DOI:** 10.1186/s40001-023-01109-3

**Published:** 2023-04-08

**Authors:** Chadanat Noonin, Tanakorn Itsaranawet, Visith Thongboonkerd

**Affiliations:** 1 Medical Proteomics Unit, Research Department, Faculty of Medicine Siriraj Hospital, Mahidol University, 6th Floor - SiMR Building, 2 Wanglang Road, Bangkoknoi, 10700 Bangkok, Thailand; 2 Biological Sciences Program, Mahidol University International College, Nakhon Pathom, 73170 Thailand

**Keywords:** COM crystal, Fibrogenesis, Kidney stone, Myofibroblast, Renal fibrosis, Renal interstitium, Renal tubules, Secreted products

## Abstract

**Background:**

Kidney stone disease (KSD) is commonly accompanied with renal fibrosis, characterized by accumulation and reorganization of extracellular matrix (ECM). During fibrogenesis, resident renal fibroblasts are activated to become myofibroblasts that actively produce ECM. However, such fibroblast–myofibroblast differentiation in KSD remained unclear. Our present study thus examined effects of secreted products (secretome) derived from proximal (HK-2) vs. distal (MDCK) renal tubular cells exposed to calcium oxalate monohydrate (COM) crystals on activation of renal fibroblasts (BHK-21).

**Methods:**

HK-2 and MDCK cells were treated with 100 µg/ml COM crystals under serum-free condition for 16 h. In parallel, the cells maintained in serum-free medium without COM treatment served as the control. Secretome derived from culture supernatant of each sample was mixed (1:1) with fresh serum-free medium and then used for BHK-21 culture for another 24 h.

**Results:**

Analyses revealed that COM-treated-HK-2 secretome significantly induced proliferation, caused morphological changes, increased spindle index, and upregulated fibroblast-activation markers (F-actin, α-SMA and fibronectin) in BHK-21 cells. However, COM-treated-MDCK secretome had no significant effects on these BHK-21 parameters. Moreover, level of transforming growth factor-β1 (TGF-β1), a profibrotic factor, significantly increased in the COM-treated-HK-2 secretome but not in the COM-treated-MDCK secretome.

**Conclusions:**

These data indicate, for the first time, that proximal and distal tubular epithelial cells exposed to COM crystals send different messages to resident renal fibroblasts. Only the secretome derived from proximal tubular cells, not that from the distal cells, induces renal fibroblast activation after their exposure to COM crystals. Such differential effects are partly due to TGF-β1 secretion, which is induced by COM crystals only in proximal tubular cells.

## Introduction

Kidney stone disease (KSD) is commonly associated with chronic kidney disease (CKD) [1]. A meta-analysis [2] and cohort studies [3] have indicated that KSD patients have a higher CKD risk. An increase in urinary excretion of oxalate (an inducer of KSD) also serves as a risk factor for CKD development [4]. High-oxalate state and calcium oxalate monohydrate (COM) crystals are known to cause oxidative stress and epithelial–mesenchymal transition (EMT) in distal renal tubular epithelial cells [5–9]. Epithelial cells with EMT lose their epithelial characteristics, gain mesenchymal phenotypes, and become myofibroblasts, which actively produce extracellular matrix (ECM) components and fibrotic factors [7, 9–11]. This process is one among others that can cause renal fibrosis, which is a characteristic of CKD [12, 13]. Nevertheless, in vivo lineage-tracing study has revealed that EMT contributes merely 5% to myofibroblast pool in the kidney [13, 14]. Indeed, the majority of renal myofibroblasts are derived from resident interstitial fibroblasts (approximately 50%) that differentiate and proliferate upon activation [13, 14].

In KSD patients, high-oxalate and high-calcium conditions as well as COM crystals directly affect renal tubular epithelial cells [15–17]. Changes in cellular proteomes of proximal and distal tubular epithelial cells upon exposure to high-oxalate and high-calcium conditions as well as COM crystals have been reported [6, 18–24]. COM crystals also cause alterations in protein composition of basolateral secretome (secreted products) derived from distal tubular epithelial cells [24, 25]. However, there is no previous report on the effects of secretome derived from renal epithelial cells exposed to COM crystals on renal fibroblast activation. Besides, it is unclear whether proximal and distal tubular cell secretomes have similar or different effects on renal fibroblast activation. Therefore, this study aimed to investigate whether secretome derived from renal tubular epithelial cells exposed to COM crystals would activate renal fibroblasts. Furthermore, the effects of secretomes derived from proximal and distal tubular cells on renal fibroblast activation were compared.

## Materials and methods

### Cell culture

All cell lines used in this study were from ATCC (Manassas, VA). HK-2 proximal tubular epithelial cells were cultured in Dulbecco’s modified Eagle medium (DMEM) (Gibco; Grand Island, NY), whereas MDCK distal tubular epithelial cells and BHK-21 renal fibroblasts were cultured in Eagle’s minimum essential medium (MEM) (Gibco). The culture media were supplemented with 10% (*v*/*v*) heat-inactivated fetal bovine serum (FBS) (Gibco), streptomycin (60 µg/ml) (Sigma-Aldrich; St. Louis, MO) and penicillin G (60 U/ml) (Sigma-Aldrich). The cells were maintained at 37 °C in a humidified incubator supplied with 5% CO_2_.

### COM crystal preparation

COM crystals were prepared as described previously [26, 27]. Briefly, 10 mM CaCl_2_·2H_2_O (Merck; Branchburg, NJ) and 1 mM Na_2_C_2_O_4_ (Sigma-Aldrich) solutions were prepared in crystallization buffer containing 90 mM NaCl (Bio Basic; Toronto, Canada) and 10 mM Tris (Affymetrix inc.; Cleveland, OH) (pH 7.4). An equal volume of 10 mM CaCl_2_·2H_2_O and 1 mM Na_2_C_2_O_4_ was mixed, and the mixture was incubated overnight at 25 °C. The formed COM crystals were harvested by centrifugation at 2000 ×*g* for 5 min. The crystals were washed three times with methanol and allowed to air-dry. Typical morphology of the COM crystals was confirmed under Nikon Eclipse Ti-S inverted phase-contrast light microscope (Nikon; Tokyo, Japan).

### COM crystal treatment

HK-2 and MDCK cells (approximately 1.5 × 10^5^ cells/well) were seeded in each well of 6-well plate (Corning Inc.; Corning, NY) with FBS-containing medium. After 18-h incubation, the culture medium was removed, and the cells were washed three times with PBS followed by one wash with FBS-free medium. The cells were then maintained in FBS-free medium with or without 100 µg/ml COM crystals for 16, 24, or 48 h. At the indicated time-points, the cells were subjected to cell death analysis as follows.

### Quantitative analysis of cell death by flow cytometry

Cell death assay was performed using fluorescein isothiocyanate (FITC)-conjugated annexin V (BD Biosciences; San Jose, CA) and propidium iodide (BD Biosciences) co-staining as described previously [28, 29]. At the indicated time-points, culture supernatant containing floating cells were collected. The adhered HK-2 and MDCK cells were trypsinized by 0.05% trypsin in 0.53 mM EDTA/PBS and 0.1% trypsin in 2.5 mM EDTA/PBS, respectively. The detached cells were collected and combined with the floating cells. The combined cells were pelleted by centrifugation at 500 ×*g* and 4 °C for 5 min and washed twice with ice-cold PBS. Thereafter, the cells were incubated with FITC-conjugated annexin V diluted in annexin V binding buffer (BD Biosciences) at 25 °C in the dark for 15 min. Subsequently, propidium iodide at the final concentration of 0.2 µg/ml was added, and the stained cells were analyzed by using BD Accuri C6 flow cytometer (BD Biosciences).

### Tubular cell secretome collection and preparation

From cell death assay, 16-h incubation was selected for secretome collection from both HK-2 and MDCK cells with or without COM crystals in serum-free medium. At this time-point, the culture supernatant from each sample was collected and centrifuged at 2000 ×*g* and 4 °C for 5 min to remove cell debris and particulate matters. The cell/debris-free supernatant collected from the cells without COM treatment was termed “control secretome”, whereas that collected from the COM-treated cells was termed “COM-treated secretome”. Each of these secretomes was diluted with an equal volume of fresh FBS-free medium and used for BHK-21 cell culture as follows.

### Renal fibroblast treatment

BHK-21 cells (approximately 2.5 × 10^4^ cells/well) were seeded in each well of 24-well plate (Corning Inc., NY) with FBS-containing medium. After 18-h incubation, the culture supernatant was removed, and the cells were washed three times with PBS followed by one wash with FBS-free medium. The cells were then incubated in control or COM-treated secretome derived from either HK-2 or MDCK cells for 24 h. Note that the 24-h incubation of BHK-21 cells with secretome derived from other cells was based on conditioning in our recent study [30]. In parallel, BHK-21 cells maintained in FBS-free medium without any secretome served as the untreated control. Thereafter, these BHK-21 cells were subjected to analyses as follows.

### Total cell count

After 24-h incubation with or without secretome derived from control or COM-treated HK-2 or MDCK cells, culture supernatant containing floating BHK-21 cells were collected. The adhered BHK-21 cells were trypsinized by 0.1% trypsin in 2.5 mM EDTA/PBS and then combined with the floating cells. Total cell count was performed using a hemacytometer.

### Cell morphology and spindle index

After 24-h incubation with or without secretome derived from control or COM-treated HK-2 or MDCK cells, morphology of BHK-21 cells was examined and imaged under the Nikon Eclipse Ti-S inverted phase-contrast light microscope. Cell boundary was manually determined using NIS-Elements D V.4.11 software (Nikon), whereas length and width of each cell were automatically measured by this software. Spindle index [31, 32] was calculated from at least 100 cells in ≥ 10 random high-power fields (HPFs) per each sample by using the following formula:

1 $${\text{Spindle index = }}{{\text{length of each cell}} \mathord{\left/ {\vphantom {{\text{length of each cell}} {\text{width of each cell}}}} \right. \kern-0pt} {\text{width of each cell}}}.$$

### F-actin, α-smooth muscle actin (α-SMA) and fibronectin immunofluorescence stainings

Approximately 2.5 × 10^4^ BHK-21 cells were seeded on a coverslip placed in each well of 24-well plate (Corning Inc.). The cells were maintained and treated as aforementioned. After 24-h incubation with or without secretome derived from control or COM-treated HK-2 or MDCK cells, BHK-21 cells were subjected to F-actin and immunofluorescence stainings as described previously [33, 34]. Briefly, the cells were fixed with 4% paraformaldehyde at 25 °C for 15 min followed by permeabilization with 0.1% Triton X-100 in PBS at 25 °C for 15 min. Thereafter, the cells were incubated with 1% bovine serum albumin (BSA) (Sigma-Aldrich) in PBS at 25 °C for 30 min to block non-specific binders. For F-actin staining, the cells were then incubated with Oregon Green 488-conjugated phalloidin (Invitrogen; Eugene, OR) (1:50) mixed with Hoechst dye (Sigma-Aldrich) (a nuclear counter stain) (1:1000) in 1% BSA/PBS at 37 °C for 1 h. For immunofluorescence stainings, the cells were incubated with mouse monoclonal anti-α-SMA antibody (Santa Cruz Biotechnology; Santa Cruz, CA) or mouse monoclonal anti-fibronectin antibody (Santa Cruz Biotechnology) (both were diluted 1:50 in 1% BSA/PBS) at 4 °C overnight. After three washes with PBS, the cells were incubated with Alexa Flour 488-conjugated anti-mouse IgG (Invitrogen) (1:500 in 1% BSA/PBS containing 1:1000 Hoechst dye) at 37 °C for 1 h.

After stainings, the cells were washed three times with PBS, and the coverslip was mounted on a glass slide using 50% glycerol in PBS. The cells were finally examined and imaged under a fluorescence microscope (Eclipse 80i) (Nikon). Fluorescence intensity data were analyzed from at least 100 cells in ≥ 10 random HPFs per each sample using NIS-Elements D V.4.11 (Nikon).

### Measurement of transforming growth factor-β1 (TGF-β1) in HK-2 and MDCK secretomes

Secretome derived from control or COM-treated HK-2 or MDCK cells (prepared as described above) was subjected to dialysis against deionized water followed by lyophilization. The lyophilized secretory proteins were dissolved in an ELISA coating buffer containing 15 mM NaCO_3_ and 30 mM NaHCO_3_ (pH 9.6), and protein concentration in each sample was measured by using Bio-Rad Protein Assay (Bio-Rad Laboratories; Hercules, CA). An equal amount (10 µg) of proteins from each sample was used to coat surface of each well of 96-well ELISA plate (Nunc; Roskilde, Denmark) at 4 °C overnight. Thereafter, excessive proteins were removed by washing with 0.05% Tween-20 in PBS, and 5% BSA in PBS was added into each well and incubated at 25 °C for 2 h to block non-specific bindings. After removing 5% BSA/PBS, each well was incubated with mouse monoclonal anti-TGF-β1 antibody (Santa Cruz Biotechnology) (diluted 1:50 in 0.1% BSA/PBS) at 25 °C for 2 h. After washing, each well was further incubated with rabbit anti-mouse secondary antibody conjugated with horseradish peroxidase (Sigma-Aldrich) at 25 °C for 2 h. After another wash, the reaction substrate containing 3.3 mM ortho-phenylenediamine dihydrochloride (Sigma-Aldrich), 0.012% H_2_O_2_, 35 mM citric acid, and 100 mM Na_2_HPO_4_ (pH 5) was added and incubated at 25 °C for 15 min in the dark. The color reaction was stopped by using 2 M H_2_SO_4_. Optical density of each sample was measured at λ492 nm using an ELISA plate reader (EZRead 400) (Biochrom; Cambridge, UK).

### Statistical analysis

All quantitative data were obtained from three independent experimental sets with different biological replicates and are reported as mean ± SD. Multiple comparisons were performed using ANOVA with Tukey’s post hoc test (for dataset with normal distribution) or Dunn’s test (for dataset without normal distribution). Comparisons between two groups of variables were performed using unpaired *t*-test (after confirming the normal distribution). Statistically significant difference was considered when the degree of difference was > 15% and *p* value was < 0.01.

## Results

### Optimal time-point for COM treatment and secretome collection from tubular cells

**Fig. 1:**
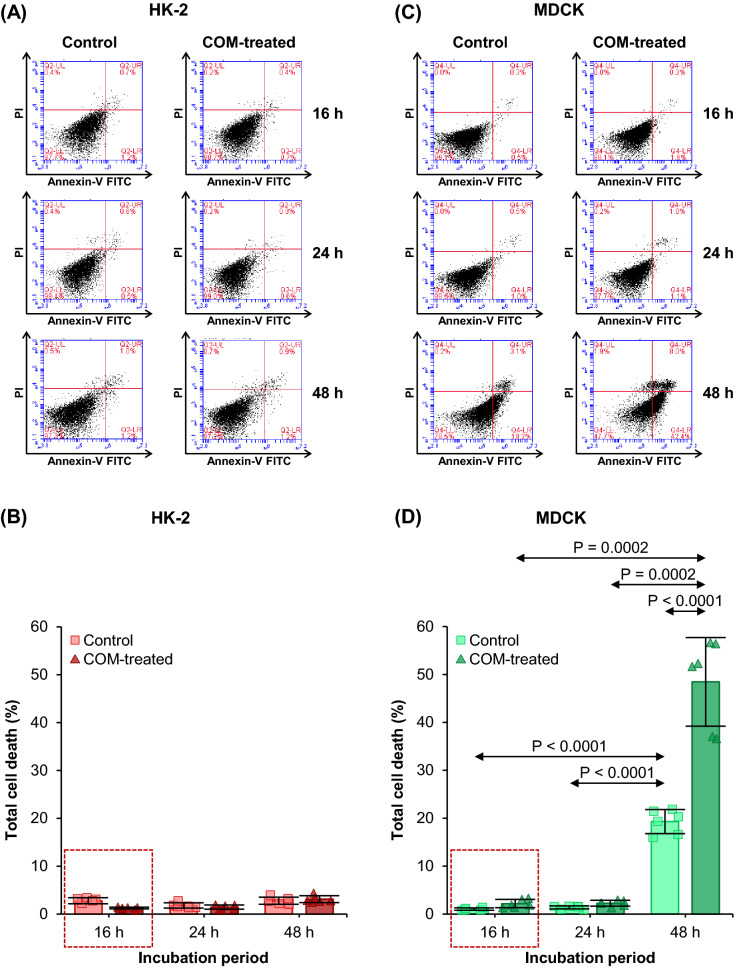
Optimal time-point for COM treatment and secretome collection from tubular cells. HK-2 and MDCK cells were incubated with 100 µg/ml COM crystals in FBS-free medium for 16, 24 and 48 h. The cells maintained in FBS-free medium without COM crystals served as the control. At the indicated time-point, the cells were stained with FITC-conjugated annexin V and propidium iodide, and cell death was quantified by flow cytometry. **(A)** and **(C):** The scatter plots showing FITC-conjugated annexin V and propidium iodide signals in HK-2 and MDCK cells, respectively. All dots that appear outside bottom-left quadrant represent the dead cells. **(B)** and **(D):** Quantitative analysis of cell death in HK-2 and MDCK cells, respectively. Each bar represents mean ± SD of measurements in each dataset (derived from three independent experiments using different biological replicates). Only significant *p* values are labeled. The red rectangle indicates the time-point chosen for secretome preparation throughout this study

The first step of this study was to define the optimal time-point for COM treatment and secretome collection from tubular cells under serum-free condition. The cells were incubated in FBS-free medium with or without 100 µg/ml COM crystals for 16, 24, or 48 h. At the indicated time-points, cell death was quantified by flow cytometry. HK-2 had < 4% cell death at all time-points for both control and COM-treated conditions. No significant difference was observed between control and COM-treated HK-2 cells at each time-point (Fig. 1A, B). Similarly, MDCK had < 4% cell death at 16 and 24 h for both control and COM-treated conditions, without significant difference between the two conditions at each time-point (Fig. 1C, D). However, cell death dramatically increased at 48 h for both conditions and was greater in the COM-treated MDCK cells. Although there was no significant increase in cell death at 24-h time-point, we selected 16-h as the optimal time-point for treatment of HK-2 and MDCK cells with COM crystals in serum-free medium prior to secretome collection to minimize the effects of waste products that might occur. In addition, the 16-h incubation agreed with several of our previous secretome studies on various cells cultured under the serum-free condition [30, 35–37].

### Effect of tubular cell secretome on renal fibroblast proliferation

**Fig. 2:**
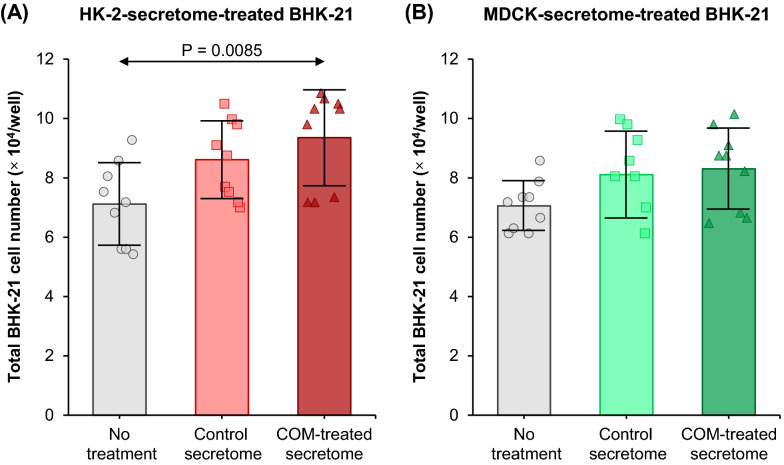
Effect of tubular cell secretome on renal fibroblast proliferation. After 24-h incubation with or without secretome derived from control or COM-treated HK-2 **(A)** or MDCK cells **(B)**, number of BHK-21 cells was counted. Each bar represents mean ± SD of measurements in each dataset (derived from three independent experiments using different biological replicates). Only significant *p* values are labeled

Increase in cell proliferation is one of the characteristics for fibroblast activation. After 24-h incubation with or without secretome derived from control or COM-treated HK-2 or MDCK cells, number of BHK-21 cells was counted. With HK-2 secretome treatment, there was no significant difference in BHK-21 cell number by the control secretome compared with the untreated condition. However, the COM-treated-HK-2 secretome significantly increased the BHK-21 cell number (Fig. 2A). With MDCK secretome treatment, there were no significant differences observed among all conditions (Fig. 2B).

### Effect of tubular cell secretome on renal fibroblast morphology and spindle index

**Fig. 3:**
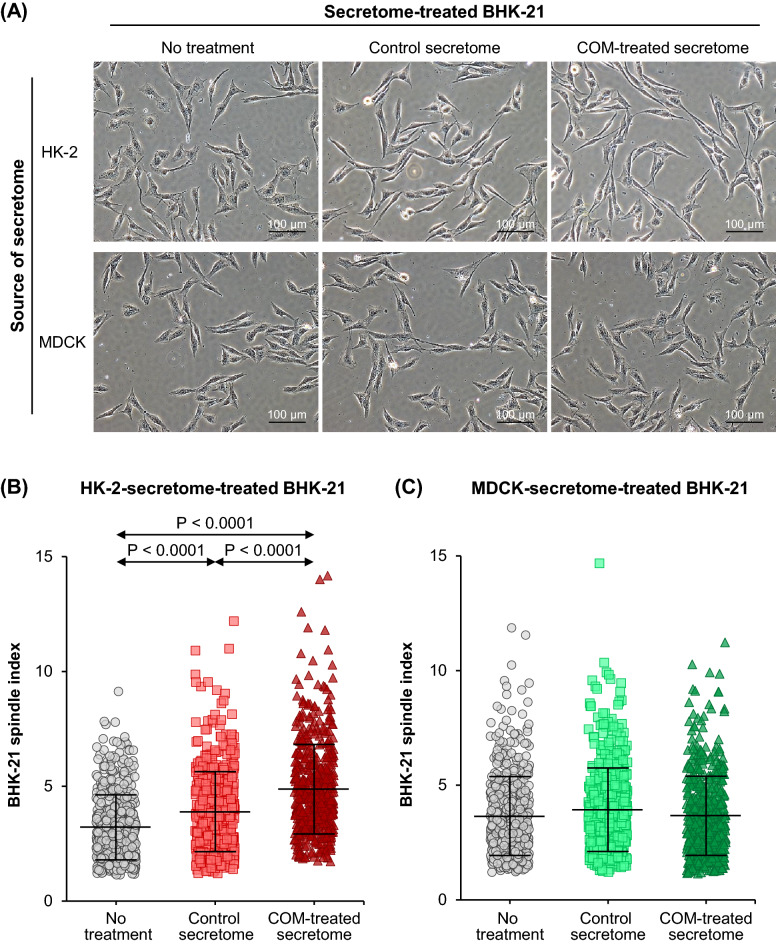
Effect of tubular cell secretome on renal fibroblast morphology and spindle index. After 24-h incubation with or without secretome derived from control or COM-treated HK-2 or MDCK cells, BHK-21 cell morphology was examined and spindle index was calculated using *Formula *1 (see “Materials and methods”). **(A):** Micrographs of BHK-21 cells. **(B)** and **(C)**: Spindle index of the BHK-21 cells treated with secretome derived from HK-2 and MDCK cells, respectively, calculated from at least 100 cells in ≥ 10 random high-power fields (HPFs) per each sample. Each bar represents mean ± SD of measurements in each dataset (derived from three independent experiments using different biological replicates). Only significant *p* values are labeled

Elongation of the fibroblast morphology is one of the parameters indicating activation/differentiation of the resident fibroblasts to myofibroblasts. After 24-h incubation with or without secretome derived from control or COM-treated HK-2 or MDCK cells, morphology of the BHK-21 was examined and spindle index was calculated using Formula 1 (see “Materials and methods”). With HK-2 secretome treatment, difference in BHK-21 morphology was observed among all three conditions. The cells were elongated after treatment with the control-HK-2 secretome compared with the untreated condition, and even longer by the COM-treated-HK-2 secretome (Fig. 3A). Spindle index significantly increased in BHK-21 cells cultured with the control-HK-2-secretome and was markedly increased by the COM-treated-HK-2 secretome as compared with the untreated condition (Fig. 3B). The COM-treated-HK-2 secretome caused significantly greater spindle index as compared with the control-HK-2 secretome (Fig. 3B). With MDCK secretome treatment, the morphology of BHK-21 looked unchanged among the three conditions. There were no significant differences in spindle index of BHK-21 cells observed among all these conditions (Fig. 3C).

### Effect of tubular cell secretome on expression of fibroblast-activation markers

**Fig. 4:**
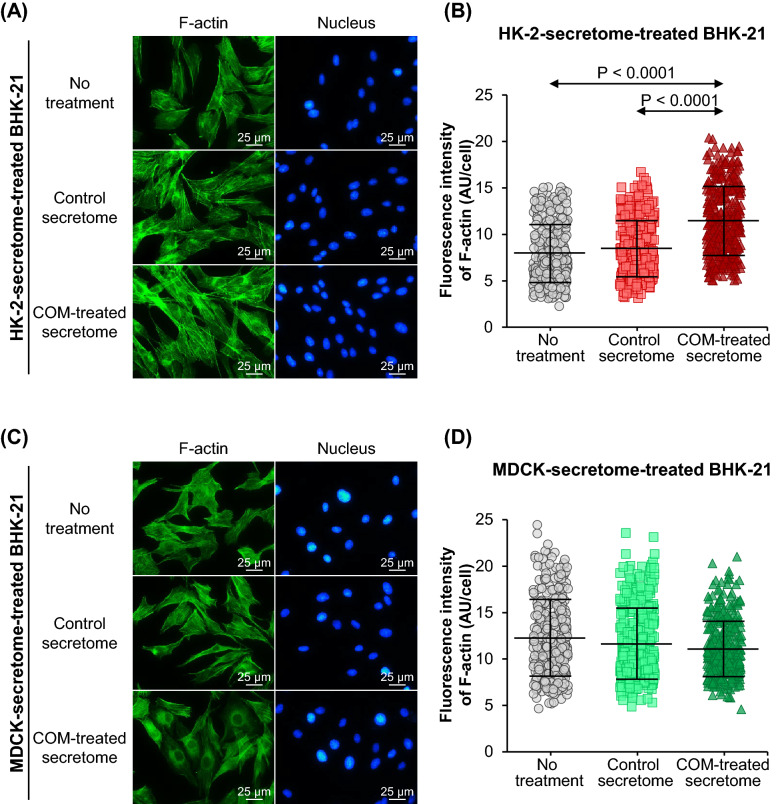
Effect of tubular cell secretome on F-actin expression in renal fibroblasts. After 24-h incubation with or without secretome derived from control or COM-treated HK-2 or MDCK cells, F-actin expression in BHK-21 cells was examined by fluorescence staining using Oregon Green 488-conjugated phalloidin. **(A)** and **(C):** Fluorescence micrographs of F-actin (in green) and nuclei (in blue) in BHK-21 cells treated with secretome derived from HK-2 and MDCK cells, respectively. **(B)** and **(D):** Fluorescence intensity data were analyzed from at least 100 cells in ≥ 10 random high-power fields (HPFs) per each sample. Each bar represents mean ± SD of measurements in each dataset (derived from three independent experiments using different biological replicates). Only significant *p* values are labeled. *AU*  arbitrary unit.

After 24-h incubation with or without secretome derived from control or COM-treated HK-2 or MDCK cells, expression levels of F-actin, α-smooth muscle actin (α-SMA) and fibronectin were evaluated in BHK-21 cells as the fibroblast-activation markers. Fluorescence staining of F-actin using phalloidin conjugated with Oregon Green 488 revealed no significant difference in F-actin expression between BHK-21 cells treated with the control-HK-2 secretome and the untreated BHK-21 cells. However, F-actin level was significantly increased by the COM-treated-HK-2 secretome (Fig. 4A, B). Treatment with the control-MDCK secretome and the COM-treated-MDCK secretome caused no significant changes of F-actin level in the BHK-21 cells as compared with the untreated cells (Fig. 4C, D).

**Fig. 5:**
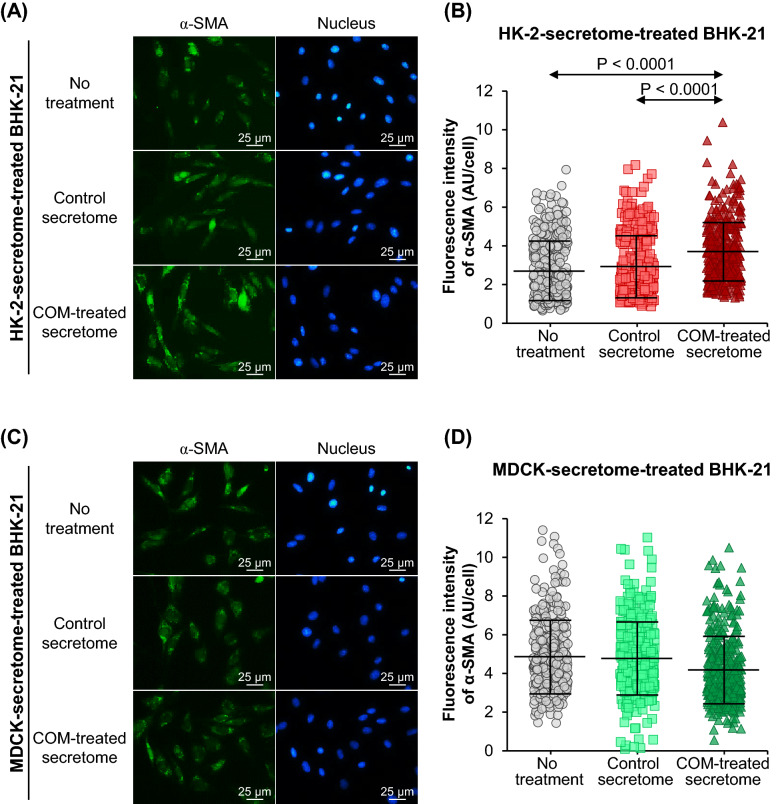
Effect of tubular cell secretome on α-SMA expression in renal fibroblasts. After 24-h incubation with or without secretome derived from control or COM-treated HK-2 or MDCK cells, α-SMA expression in BHK-21 cells was examined by immunofluorescence staining using mouse monoclonal anti-α-SMA primary antibody and Alexa Flour 488-conjugated anti-mouse IgG secondary antibody. **(A)** and **(C):** Immunofluorescence micrographs of α-SMA (in green) and nuclei (in blue) in BHK-21 cells treated with secretome derived from HK-2 and MDCK cells, respectively. **(B)** and **(D):** Fluorescence intensity data were analyzed from at least 100 cells in ≥ 10 random high-power fields (HPFs) per each sample. Each bar represents mean ± SD of measurements in each dataset (derived from three independent experiments using different biological replicates). Only significant *p* values are labeled. *AU* arbitrary unit

**Fig. 6:**
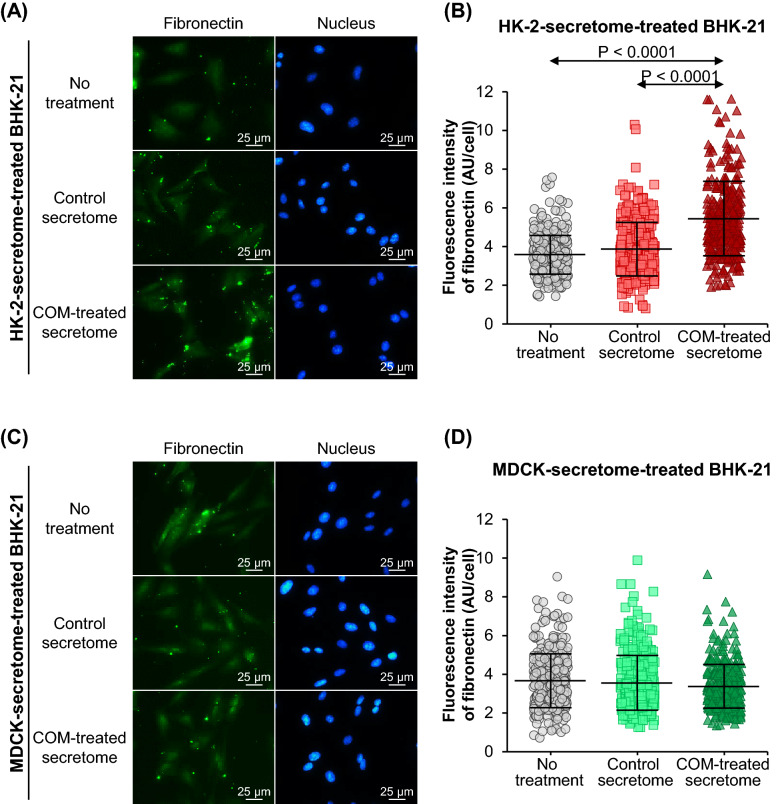
Effect of tubular cell secretome on fibronectin expression in renal fibroblasts. After 24-h incubation with or without secretome derived from control or COM-treated HK-2 or MDCK cells, fibronectin expression in BHK-21 cells was examined by immunofluorescence staining using mouse monoclonal anti-fibronectin primary antibody and Alexa Flour 488-conjugated anti-mouse IgG secondary antibody. **(A)** and **(C):** Immunofluorescence micrographs of fibronectin (in green) and nuclei (in blue) in BHK-21 cells treated with secretome derived from HK-2 and MDCK cells, respectively. **(B)** and **(D):** Fluorescence intensity data were analyzed from at least 100 cells in ≥ 10 random high-power fields (HPFs) per each sample. Each bar represents mean ± SD of measurements in each dataset (derived from three independent experiments using different biological replicates). Only significant *p* values are labeled. *AU* arbitrary unit

Similarly, immunofluorescence stainings of α-SMA and fibronectin showed no significant differences in expression levels of these two markers in BHK-21 cells treated with the control-HK-2 secretome compared with the untreated BHK-21 cells (Figs. 5A, B, 6A, B). However, levels of α-SMA and fibronectin were significantly increased by the COM-treated-HK-2 secretome (Figs. 5A, B, 6A, B). Treatment with the control-MDCK secretome and the COM-treated-MDCK secretome caused no significant changes of α-SMA and fibronectin levels in the BHK-21 cells as compared with the untreated cells (Figs. 5C, D, 6C, D).

### TGF-β1 levels in control and COM-treated HK-2 and MDCK secretomes

**Fig. 7:**
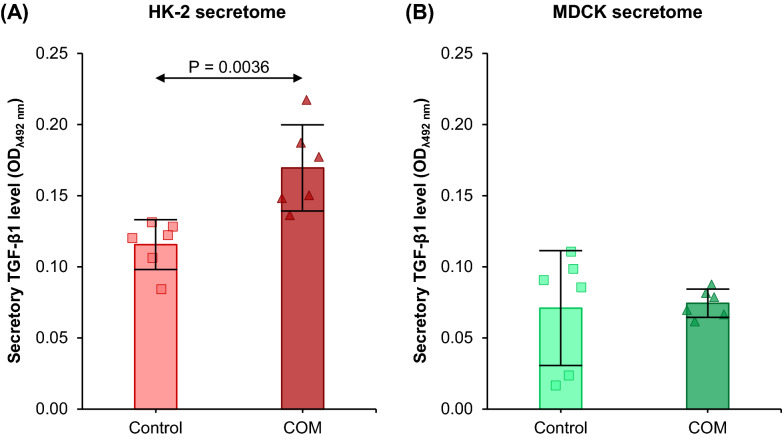
TGF-β1 levels in control and COM-treated HK-2 and MDCK secretomes. Levels of TGF-β1 in control and COM-treated HK-2 **(A)** and MDCK **(B)** secretomes were measured by ELISA. Each bar represents mean ± SD of measurements in each dataset (derived from three independent experiments using different biological replicates). Only significant *p* values are labeled

To determine the molecule responsible for differential effects of the COM-treated-HK-2 and COM-treated-MDCK secretomes, levels of TGF-β1, a profibrotic factor, were measured in these secretomes by ELISA. The results showed that COM crystals caused significant increase in secretory TGF-β1 in HK-2 cells (Fig. 7A). However, there was no significant change observed in secretory TGF-β1 level in the COM-treated-MDCK secretome as compared with the control (Fig. 7B).

## Discussion

Several stimuli can directly activate renal interstitial fibroblasts. Direct exposure to a hypoxic condition induces renal fibroblasts to be elongated with increases in spindle index and filamentous actin (F-actin) formation [38]. In addition, the fibroblast-activation markers, such as α-SMA and fibronectin, are upregulated after activation [38]. Lung fibroblasts exposed to fine particulate matter (PM_2.5_) or cancer drug (etoposide) have increased α-SMA and collagen I expression [39, 40]. Besides, secretion of a profibrotic cytokine, TGF-β1, is enhanced after fibroblast activation [39, 40].

Alternatively, fibroblasts can be activated without direct exposure to the primary stimuli, i.e., by exposure to secreted products (secretome) of other cells receiving such primary stimuli. COM-stimulated macrophages release secretome and exosomes containing proteins different from their naïve or unstimulated form [30, 41, 42]. Treating renal fibroblasts with secretome obtained from the COM-stimulated macrophages results in renal fibroblast activation [30]. Their activation can be determined by upregulation of F-actin, α-SMA, fibronectin and matrix metalloproteinases (MMPs) [30]. Additionally, exosomes derived from renal epithelial cells under a high-glucose condition can activate renal fibroblasts [43]. Moreover, secretome derived from senescent renal epithelial cells stimulates proliferation and expression of α-SMA, fibronectin and collagen I in lung fibroblasts [10]. Co-culture of lung fibroblasts with chemical-induced-senescent lung epithelial cells results in higher degree of cell proliferation and enhanced expression of α-SMA, collagen I and vimentin [44]. This stimulatory effect is triggered by Nanog protein expression in fibroblasts induced by senescence-associated secretory phenotype of the senescent epithelial cells [44].

Interstitial fibrosis is one of the pathologic features of progressive KSD and is a sign of CKD development [45, 46]. The cells that are responsible for fibrogenesis are myofibroblasts, which are rarely present in the normal renal tissue [47, 48]. Under the pathologic conditions, renal myofibroblasts can be derived or differentiated from several cell types. A small portion of myofibroblasts is originated from activated pericytes, mesenchymal stem cells, epithelial cells undergoing epithelial–mesenchymal transition (EMT), and endothelial cells undergoing endothelial–mesenchymal transition (EndMT) [14, 47]. Actually, the majority of renal myofibroblasts are derived from resident interstitial fibroblasts after activation [13, 49]. Upregulation of fibrotic or fibroblast-activation markers, such as α-SMA, fibronectin and collagen I, has been detected in mouse kidney with calcium oxalate (CaOx) crystal-induced CKD [50, 51]. The upregulation of these fibrotic markers in the kidney is commonly associated with induction of immune responses and inflammatory cascades [52–54], suggest the interplay among many cell types in KSD-induced CKD.

The crosstalk among different cell types in response to CaOx crystal exposure is well documented. Co-culture of macrophages with COM-treated tubular epithelial cells leads to macrophage activation with high expression of TLR4 and IRF-1 [55]. Exosomes derived from macrophages treated with COM crystals stimulate IL-8 secretion from renal epithelial cells [42]. These exosomes also induce migration of monocytes and T-cells [41]. Additionally, secretome derived from the COM-treated macrophages activates renal fibroblasts with increased levels of F-actin, α-SMA, fibronectin and MMPs [30]. In concordance, our present study revealed the crosstalk of the COM-treated epithelial cells from proximal and distal tubules with renal fibroblasts.

Obviously, our data showed that the COM-treated proximal tubular cells secreted products (secretome) that induced renal fibroblast activation. The renal fibroblasts became more proliferative and elongated with increased spindle index. Additionally, the actin stress fiber (F-actin) and other fibroblast-activation markers (α-SMA and fibronectin) were upregulated. Nonetheless, these activation phenotypes and markers were not induced by secretome derived from the COM-treated distal tubular cells. These different effects of proximal vs. distal tubular cell secretomes on renal fibroblast activation were most likely due to their differential secretome compositions. One of such differential compositions was TGF-β1 level, which increased only in the COM-treated proximal tubular cell secretome, not in the COM-treated distal tubular cell secretome. This data was consistent with a recent report showing differential degrees of TGF-β1 secretion by HK-2 and MDCK cells treated with urinary exosomes contaminated by bacterial extracellular vesicles [56].

Moreover, it has been reported that different renal tubular segments secrete differential amounts of extracellular vesicles [16]. Proximal tubules of KSD patients and non-KSD subjects secrete greater number of extracellular vesicles containing MCP-1 and neutrophil gelatinase-associated lipocalin (NGAL) than distal tubules [16]. Additionally, each tubular segment secretes different biomarkers, which can be detected in the urine and be used to localize the injury site [57]. For instance, the increased urinary level of kidney injury molecule-1 (KIM-1), a marker for proximal tubular injury, is associated with acute kidney injury (AKI) and CKD development [58].

Another possibility of their differential fibroblast-activation effects is that these tubular epithelial cells differently respond to COM crystal exposure. Differential responses of proximal vs. distal tubular epithelia to other stimuli have been reported. After ischemic reperfusion, actin dysregulation occurs faster in proximal than distal tubular cells [59]. Both proximal and distal tubular cells express calcium-sensing receptor (CaSR) [60]. Nonetheless, CaSR localizes on apical side of the proximal cells, but appears on basolateral side of the distal ones [61, 62]. Therefore, CaSR in proximal tubules responds to changes of intratubular (luminal) calcium level and regulates transport of calcium, phosphate and citrate across apical membranes. On the other hand, distal tubular CaSR reacts to and regulates changes in serum/interstitial calcium level [63, 64]. Although our data exhibited differential secretory TGF-β1 levels in response to COM crystals between proximal and distal cells, the more precise mechanisms underlying differential effects of the COM-treated proximal vs. distal tubular cell secretomes on renal fibroblast activation deserve further investigations and elucidations.

## Conclusions

This is the first report for the crosstalk between renal epithelial cells from different tubular segments and renal fibroblasts. Taken together, the results in this study have clearly shown that the COM-treated proximal tubular cell secretome effectively activates renal interstitial fibroblasts. However, the secretome derived from the COM-treated distal tubular cells has no significant effect on renal fibroblast activation. These data suggest that proximal renal tubular cells possibly play more important roles in promoting COM crystal-induced renal fibrosis. Such differential effects are partly due to TGF-β1 secretion, which is induced by COM crystals only in proximal tubular cells. However, which other protein (s) or product (s) in the COM-treated proximal tubular cell secretome exert (s) such activating effects on renal fibroblasts should be further elucidated.

## Data Availability

All data generated or analyzed during this study are included in this article and are also available from the corresponding author on reasonable request.
